# 

**Published:** 1887-04

**Authors:** 


### BOOKS AND PAMPHLETS RECEIVED.

STUDIES FROM THE BIOLOGICAL LABORATORY, Johns Hopkins University, edited by H. Newell Martin and W. K. Brooks. Vol. III., Nos. 8 and 9, Johns Hopkins University Circulars. Baltimore.

ANNALS ET BULLETINS SOCIETIE DE MEDECINE DE GAND. Ghent, Belgium.

PROCEEDINGS NEWPORT NATURAL HISTORY SOCIETY, 1885–86.

SCOTTISH AGRICULTURAL GAZETTE ALMANAC contains an article on Pleuro-Pneumonia by Principal Thomas Walley. Edinburgh, 1887.

PRESIDENT'S ADDRESS, TENTH ANNUAL MEETING OF THE DETROIT MEDICAL AND LIBRARY ASSOCIATION. By C. J. Lundy, A. M., M. D.

THE TRUE RELATIONS OF THE VETERINARY PROFESSION TO THE LIVE-STOCK INTERESTS OF THE STATE. By Frank S. Billings, D. V. M.

STERILITY, MANAGEMENT OF THE SECUNDINES AND REPORT ON DISEASES OF THE RECTUM. By Joseph M. Mathews, M. D. Louisville. 1886.

CLASSIFICATION OF THE MACROCHIRES. By R. W. Shufeldt, M. D.

CONTRIBUTIONS TO SCIENCE AND BIOGRAPHICAL RESUME OF THE WRITINGS OF R. W. SHUFELDT, M. D., Captain Medical Department, U. S. A., 1881–1887. L. S. Foster, New York, 1887.

THE DIETETIC ANNUAL FOR 1887. Published by Wells, Richardson & Co.

UBER WIRKUNG, therapeutischen werth und Gebrauch des neuen Karlsbader Quellsalzes, nebst dessen Beziehung, zum Karlsbader thermalwasser, Von Dr. W. Jawoiski, Wein, 1886.

COOPER MEDICAL COLLEGE, San Francisco, annual announcement, Session 1887.

ANNUAL REPORT OF MORSE DISPENSARY OF COOPER MEDICAL COLLEGE, 1886.

BIENNIAL REPORT OF THE STATE BOARD OF HEALTH OF THE STATE OF WEST VIRGINIA, 1885–'86.

